# Improving clinical diagnostic accuracy and management of False penile fractures characterizing typical clinical presentation: a systematic review and meta-analysis

**DOI:** 10.1007/s00345-023-04456-2

**Published:** 2023-06-16

**Authors:** Edoardo Agostini, Antonio Vinci, Dorian Bardhi, Fabio Ingravalle, Mario Muselli, Giulio Milanese

**Affiliations:** 1 Department of Urology, “IRCCS–INRCA” Hospital, 60127 Ancona, Italy; 2 Department of Biomedicine and Prevention, University of Rome “Tor Vergata”, 00133 Rome, Italy; 3 Hospital Health Management Area, Local Health Authority “Roma 1”, 00133 Rome, Italy; 4 Post-Graduate School of Hygiene and Preventive Medicine, University of L’Aquila, 67100 L’Aquila, Italy; 5 Department of Life, Health and Environmental Science, University of L’Aquila, Piazzale Salvatore Tommasi, 1, 67100 L’Aquila, Italy; 6 Hospital Health Management Area, Local Health Authority “Roma 6”, 00041 Albano Laziale, Italy; 7 Post-Graduate School of Urology, Polytechnic University of Marche, 60121 Ancona, Italy

**Keywords:** Penis trauma, Penis rupture, False penile fractures, Penis diagnosis

## Abstract

**Purpose:**

False penile fractures (FPF) represent a rare sexual emergency characterized by blunt trauma of penis in the absence of albuginea’s injury, with or without lesion of dorsal penile vein. Their presentation is often indistinguishable from true penile fractures (TPF). This overlapping of clinical presentation, and lack of knowledge about FPF, can lead surgeons often to proceed directly to surgical exploration without further examinations. The aim of this study was to define a typical presentation of false penile fractures (FPF) emergency, identifying in absence of “snap” sound, slow detumescence, penile shaft ecchymosis, and penile deviation main clinical signs.

**Methods:**

We performed a systematic review and meta-analysis based on Medline, Scopus and Cochrane following a protocol designed a priori, to define sensitivity of “snap” sound absence, slow detumescence and penile deviation.

**Results:**

Based on the literature search of 93 articles, 15 were included (73 patients). All patients referred pain, most of them during coitus (*n* = 57; 78%). Detumescence occurred in 37/73 (51%), and all patients described detumescence occurrence as “slow”. The results show that single anamnestic item have a high-moderate sensibility in the diagnosis of FPF, and the highest sensitive item was penile deviation (sensibility = 0.86). However, when more than one item is present, overall sensitivity increases greatly, closing to 100% (95% Confidence Interval 92–100).

**Conclusion:**

Surgeons can consciously decide between additional exams, a conservative approach, and rapid intervention using these indicators to detect FPF. Our findings identified symptoms with excellent specificity for FPF diagnosis, giving clinicians more useful tools for making decisions.

## Introduction

False penile fractures (FPF) represent a small percentage of urologic emergencies and sometimes have a presentation indistinguishable from true penile fractures (TPF). The absence or presence of tunica albuginea disruption sets the differential diagnosis between these two entities [1]. The pathogenesis of FPF is commonly considered a rupture of the dorsal superficial vein of the penis, with circumcision as a predisposing factor due to stretching of the penile skin during intercourse [2]. Coitus, manipulation, rolling in bed, or falling are the most reported causes for both [1, 3]. The main difference between TPF and FPF is that the latter can, in some cases, be managed conservatively.

Due to the analogous pathogenesis, patients are often misdiagnosed as having true penile fractures by clinicians and submitted to surgery without additional imaging, for the most part, to confirm the diagnosis [5], evaluating Buck's fascia and dorsal vein integrity [6]. This leads to many unnecessary surgeries, causing severe discomfort for patients undergoing invasive operations and avoidable costs for the public health system.

No robust data exist to date aiming to evaluate the sensitivity and specificity of symptoms and signs such as pain, “snap” or “crack” sound, deviation, detumescence speed, or ecchymosis, which can differentiate between TPF and FPF. Therefore, we decided to systematically review the literature on FPF to evaluate the sensitivity of each of these signs and symptoms. The null hypothesis (H0) is that there is no clear difference in clinical presentation between true and false penile fractures.

This research and statistical analysis aim to provide evidence for clinicians to decide between further radiological examination or immediate surgery. Hopefully, this would prevent patients from undergoing unnecessary surgery in the case of false penile fractures.

## Methods

This systematic review and meta-analysis were performed following a pre-defined protocol registered on the Open Science Framework (OSF) Registries on September 10, 2022 (https://osf.io/ybgj3) [7]. The research question was developed using the “PECO” approach; “P” stands for Patients, “E” for Exposure, “C” for Comparison, and “O” for Outcome. The PECO items were defined as follows:

Population: patients with false penile fracture;

Exposure: absence of snap sound and/or absence of penile detumescence and/or penile deviation;

Comparison: patients with no previous signs;

Outcome: surgical diagnosis of false penile fracture.

No patients were involved in the conduct of this study. Patient and public involvement were not planned or sought in this systematic review.

### Eligibility criteria

Since it was clear, after a preliminary search, that the body of evidence consists only of uncontrolled clinical observations due to the total absence of diagnostic trials on the matter, the inclusion and exclusion criteria were tailored for case series and case reports. In the biomedical literature, a case report is the description of the clinical course of one individual, which may include specific exposures, symptoms, signs, interventions, or outcomes. A case report is the smallest publishable unit in the literature, whereas a case series report aggregates individual cases in one publication [8]. Although sub-optimal, in some instances, a meta-analysis of this type of evidence can be performed [9]. Studies meeting the following criteria were included in this systematic review:

Study design: Case series or case reports;

Exposure: Study reporting cases of false penile fractures;

Publication Type: Primary studies published in peer-reviewed journals.

Studies meeting the following criteria were excluded from the systematic review:

Study design: Cross-sectional (prevalence) studies;

Publication type: Studies reporting results that have been superseded by subsequent reports from the same study (note: this includes conditions when those reports include a mix of results that have or have not been updated).

### Information sources and search strategies

The Medline, Scopus, and Cochrane databases were electronically searched on 15/09/2022 using the broadest search strategy due to the already known scarcity of evidence: (false AND penile AND fracture) OR (dorsal AND vein AND rupture). No filter or restriction was applied. It was not possible to search for MeshTerms related to penis fracture because they are not present on the National Library of Medicine site (Medline).

### Selection process, data items, and data extraction

Three different authors independently searched for articles and collected data. Studies with missing or unclear data were excluded. Disagreements were discussed by the authors and resolved through consensus or by consulting a fourth author. For every study included in the meta-analysis, the following data were retrieved:

Publication year.

Country.

Number of included patients.

Number of patients reporting no snap sound.

Number of patients reporting no detumescence.

Number of patients reporting penile deviation.

The quality of the studies was evaluated using the JBI critical appraisal checklist for case reports and case series [10].

### Effect measures

The computed effect measure was the sensitivity of each reported “Exposure” item, i.e., absence of snap sound, penile detumescence, and penile deviation. Due to the absence of a comparison in the available evidence, specificity could not be calculated. Other incidentally retrieved information from the included articles, such as the usage of ultrasonography, presence of ecchymosis, and integrity of the tunica albuginea, were not used for the meta-analysis but were considered in a narrative synthesis.

### Synthesis methods

The results have been reported both narratively and graphically and summarized in tables when appropriate. All studies meeting the inclusion criteria were selected for narrative synthesis.

For quantitative synthesis, all data from the retrieved case reports were grouped under the category “case reports.” Since quantitative synthesis involved the analysis of case series, sometimes with a 100% event rate, the Laplace succession rule was used: for each series, 2 pseudo-observations were added, one with the reported “Exposure” item and one without, assuming that no prior sensitivity distribution was known [11].

Given the data type and the involvement of different populations, a Random Effects empirical Bayes model was chosen for the meta-analysis. Statistical heterogeneity was calculated using the *I*^2^ statistic [12]. Confidence Intervals (CIs), *I*^2^, and *p* values were calculated to highlight potential precision issues. Stata^®^ SE v.17.0 and Microsoft Excel MSO 2016 were used for calculations and graph drawing.

This systematic review and meta-analysis were reported according to the 2020 Preferred Reporting Items for Systematic Reviews and Meta-Analyses (PRISMA) guidelines [13].

## Results

### Study selection and study characteristics

**Fig. 1:**
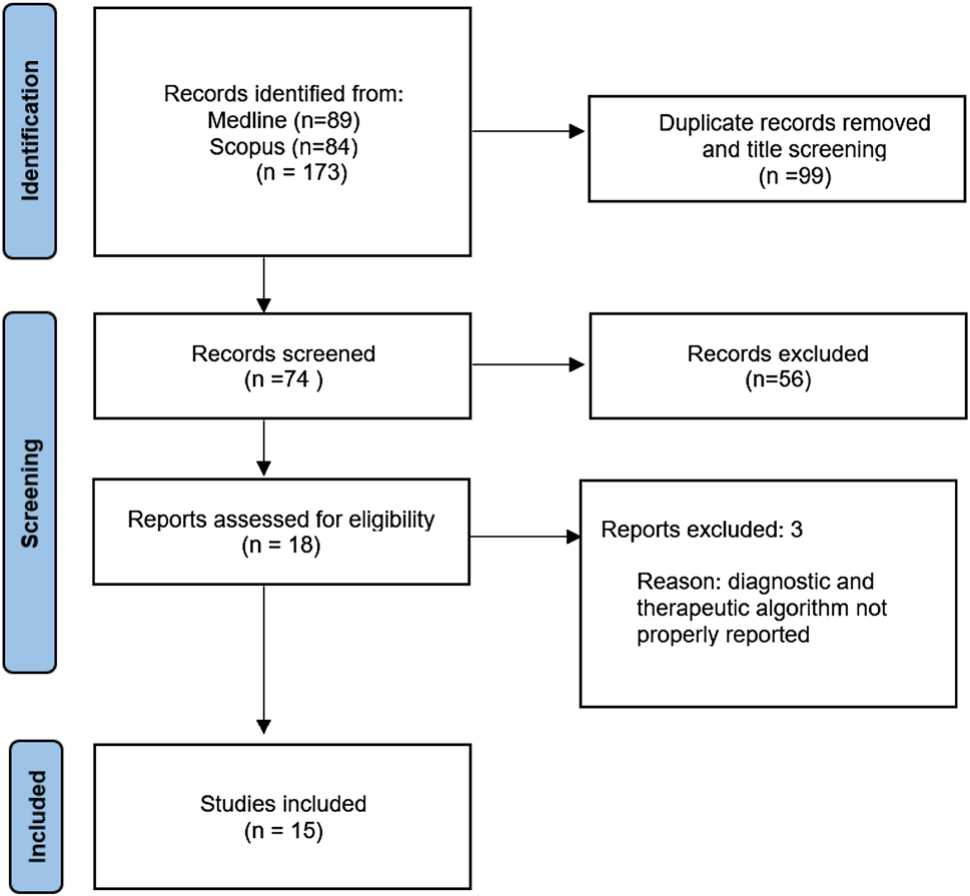
PRISMA flowchart of literature search

A flow chart of the literature screening is shown in Fig. 1. Overall, 173 studies were retrieved from databases, of which 99 were duplicates. After eliminating duplicates, 74 articles remained. After the screening sessions, 18 studies were fully evaluated, and 79 studies were excluded. Out of the 18 articles, 3 did not properly report diagnostic and therapeutic algorithms and were excluded. Finally, 15 studies were included in the systematic review, comprising a total of 73 patients. The characteristics of the included studies and a summary of the findings are presented in Table 1. The results of the searches and selection process are shown in Fig. 1.

**Table 1:** Characteristics of included studies

Author	Year	Country	Patients	Snap sound	Detumescence	Penile deviation	Ecchymosis of the shaft	Tunical defect
Feki et al. [4]	2004	Tunisia	16	5	nr	nr	16	0
Rafiei et al. [14]	2014	USA	1	0	0	0	1	0
Al Reshaid et al. [15]	2010	Saudi Arabia	1	0	1	nr	1	nr
Kurkar et al. [3]	2014	Egypt	11	2	11	0	11	0
Aminu et al. [16]	2011	UK	1	0	0	nr	1	nr
Scott et al. [6]	2021	USA	1	0	1	nr	1	nr
Eken et al. [17]	2014	Turkey	1	0	0	nr	1	0
Cabral Dias-Dilho et al. [18]	2020	Brazil	9	2	9	nr	nr	nr
Nehru-Babu et al. [19]	2009	UK	1	0	0	nr	1	nr
Bar-Yosef et al. [2]	2007	Israel	9	0	9	nr	nr	nr
El-Assmy et al. [20]	2010	Egypt	17	2	3	2	17	nr
Truong et al. [21]	2016	Australia	1	0	1	1	1	nr
Baran et al. [22]	2011	Turkey	1	0	0	nr	1	nr
Nicely et al. [23]	1992	USA	1	nr	nr	1	1	nr
Shah et al. [5]	2003	USA	2	0	2	1	2	0

*Nr* not reported

### Quality of evidence

**Fig. 2:**
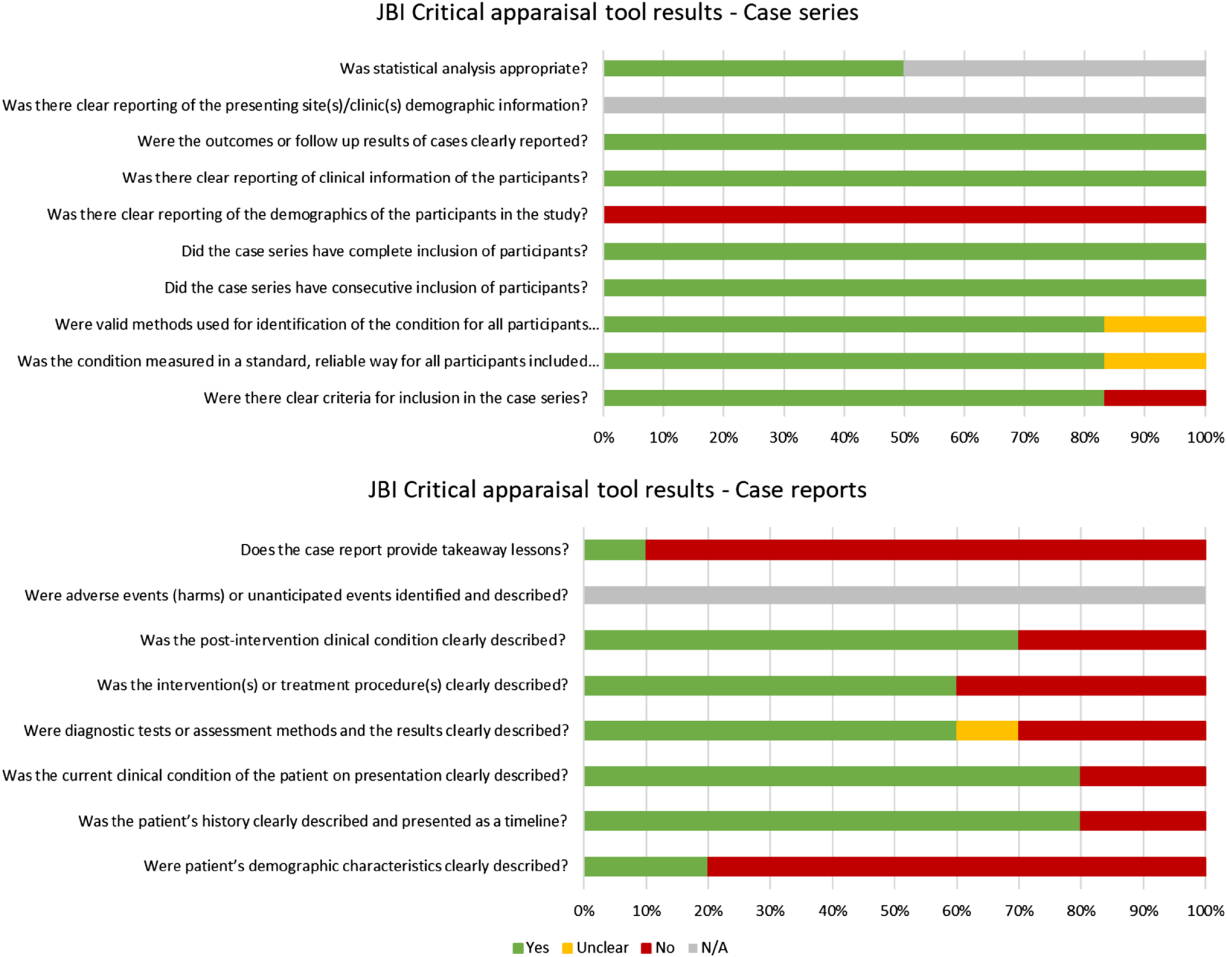
JBI critical appraisal tool

The results of the JBI critical appraisal tool are presented in Fig. 2. Case reports generally had low overall quality compared to case series, which were more complete in several key aspects.

### Results of individual studies

Fifteen articles involving 73 patients were analyzed. All patients reported experiencing pain during activities such as rolling in bed (*n* = 5; 7%), coitus (*n* = 57; 78%), or manipulation (*n* = 11; 15%). Four authors mentioned patients reporting snap sounds. Among the 73 patients, 11 (15%) reported a “cracking” sound during the presumed false penile fracture (FPF) event. Detumescence occurred in 37 out of 73 patients (51%), with 2 authors not recording this sign. All patients described the occurrence of detumescence as “slow”. Penile deviation was observable in 5 patients (7%), but without any palpable penile skin defect. Only 5 authors reported an examination of the albuginea's integrity during the clinical assessment. Ecchymosis of the penile shaft was observed in 55 patients (75%) upon inspection. Surgical exploration was performed in all patients.

**Fig. 3:**
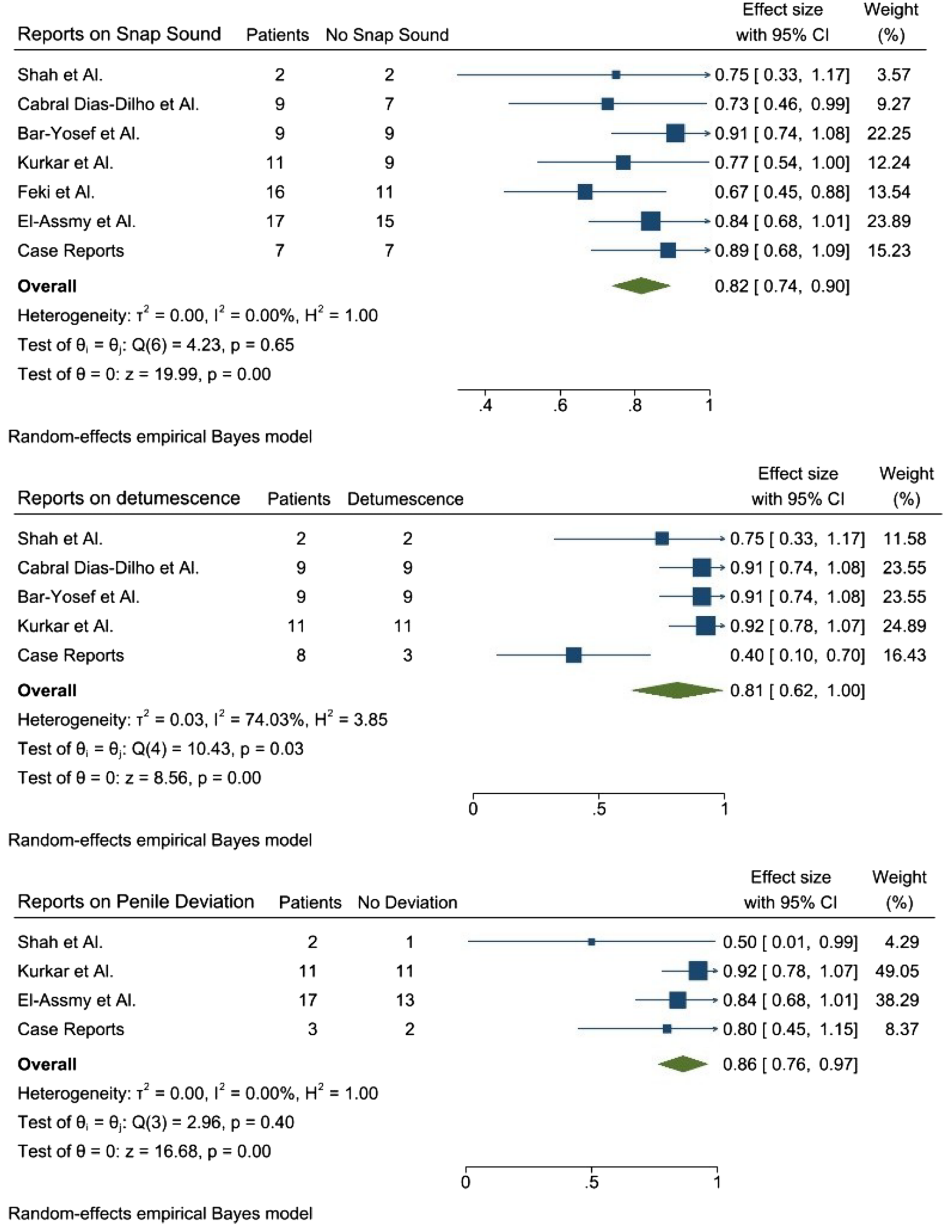
Sensitivity Forest plot for snap sound absence, penile detumescence, and penile deviation

The sensitivity for the absence of a “snap” sound was 82%, which increased to 97% when combined with either slow detumescence or penile deviation. The sensitivity for slow detumescence alone was 81%, while for penile deviation alone, it was 86%. Overall, the results indicated that the presence of multiple signs increased the sensitivity significantly, reaching close to 100% (95% Confidence Interval: 92–100%). Meta-analysis results were summarized with three forest plots (Fig. 3).

## Discussion

False penile fractures (FPF) represent an urgent condition in andrology, but only a small percentage requires surgery, leading to unnecessary or harmful procedures [24]. However, there is currently no strong evidence to guide clinicians in their decision-making process. Our research identified the most common signs and symptoms in FPF reports and series [3, 5, 20]: absence of a “snap” sound, slow detumescence, penile deviation, ecchymosis of the shaft, and absence of tunical defects.

The cracking or snapping sound is typically considered crucial in diagnosing true penile fractures (TPF) and indicates a rupture of the tunica albuginea. According to the literature, this sound correlates with a true fracture in 72–84% of cases, increasing to 92% when accompanied by immediate detumescence [18, 25]. Our meta-analysis showed that the absence of a cracking sound has a sensitivity of 82% in FPF, rising to 96% when combined with slow detumescence. However, a series of 11 fractured penises reported a 55% incidence of snapping sound in TPF, raising concerns about the reliability of this sign [26].

Detumescence is always described as “slow” in FPF, with an overall sensitivity of 81%. In contrast, in TPF, it is always described as “quick” and is reported in 100% of patients [25]. Penile ecchymosis, resulting from blood spread through intact tunica layers, has a sensitivity of 100% in our study. Differentiating from TPF, where a large hematoma with a “rolling sign” is considered pathognomonic, FPF often presents with ecchymosis limited to the shaft [27]. Uncommon shapes of penile ecchymosis associated with FPF include rectangular-shaped ecchymosis in the suprapubic area with swelling of the distal penile skin, indicating complete transection of the dorsal penile vein [16]. A butterfly hematoma is described in a small percentage of TPF cases [25].

Tunical defects were not included in our statistical analysis due to limited reporting by authors. However, tunic defects are reported in only 34% of TPF cases [25]. Albuginea's injury results in penile deviation contralateral to the injury, causing an eggplant deformity [28]. Penile angulation is reported in 64% of TPF patients but is also described in FPF, with a sensitivity of 86%. When combined with the absence of snap and detumescence, the sensitivity rises to 97% for each combination of signs.

Ultrasound is an additional tool for clinicians, providing an inexpensive and non-invasive means of investigation. Although lacking a pathognomonic pattern, ultrasound can increase awareness of FPF by showing intact tunica albuginea, dorsal vein thrombosis, and/or hematoma with ruptured or dilated veins [6, 14]. Some authors suggest performing ultrasound only in cases of unusual presentation and monitoring suspected FPF patients for clinical stability. Surgery may be considered if the hematoma worsens or symptoms/blood exams deteriorate [14, 26, 29].

The use of penile cavernosography for imaging remains controversial due to limitations in visualizing small cavernosal defects and the risk of penile fibrosis from contrast medium leakage. Additionally, it is an invasive technique associated with a high radiation exposure, which is considered detrimental in younger patients [26]. Magnetic resonance imaging is the gold standard for evaluating albuginea injuries, but its high costs and long execution times discourage routine use in emergency settings [26, 30].

Surgeons may be concerned about the risk of complications when opting for watchful waiting instead of immediate surgery. While TPF requires prompt management, only Al-Reshaid et al. reported a case of penile abscess and necrotizing fasciitis in a not recognized FPF [15].

## Conclusions

This study is the first to systematically review studies on false penile fractures (FPF) and provide a meta-analysis to depict the typical clinical presentation. Surgeons often fail to recognize FPF and perform unnecessary operations due to the overlap in pathogenesis with true penile fractures (TPF). We identified the absence of a cracking sound, slow detumescence, penile deviation, and ecchymosis of the shaft were identified as highly sensitive signs for diagnosing FPF. Employing these signs to suspect FPF, eventually associated with ultrasound examination, surgeons acquire an additional tool helpful for decision-making when managing blunt penile traumas suspected of fracture.

The main limitation of the study is the lack of diagnostic randomized controlled trials (RCTs), and we had to rely on case reports and case series to derive evidence. These limitations could not be overcome but were represented in the study quality appraisal. Additionally, the specificity of identified signs could not be assessed due to the absence of a control group for false positives, and no receiver operating characteristic (ROC) curve could be proposed.

## Data Availability

Data sharing is not applicable to this article as no datasets were generated or analysed during the current study.

## References

[1] Gaspar SS, Dias JS, Martins F, Lopes TM (2015). Sexual urological emergencies. Sex Med Rev.

[2] Bar-Yosef Y, Greenstein A, Beri A, Lidawi G, Matzkin H, Chen J (2007). Dorsal vein injuries observed during penile exploration for suspected penile fracture. J Sex Med..

[3] Kurkar A, Elderwy AA, Orabi H (2014). False fracture of the penis: Different pathology but similar clinical presentation and management. Urol Ann.

[4] Feki W, Derouiche A, Belhaj K, Ouni A, Ben Mouelhi S, Ben Slama MR (2007). False penile fracture: report of 16 cases. Int J Impot Res.

[5] Shah DK, Paul EM, Meyersfield SA, Schoor RA (2003). False fracture of the penis. Urology.

[6] Scott SE, Langenohl R, Crisostomo-Wynne T, Kang C (2021). Penile dorsal vein rupture identified by emergency department ultrasound. Clin Pract Cases Emerg Med.

[7] Vinci A, Bardhi D, Agostini E, Muselli M (2022) Protocol. Available at: https://osf.io/ybgj3/

[8] Grimes DA, Schulz KF (2002). Descriptive studies: what they can and cannot do. The Lancet.

[9] Murad MH, Sultan S, Haffar S, Bazerbachi F (2018). Methodological quality and synthesis of case series and case reports. BMJ EBM.

[10] Gagnier JJ, Kienle G, Altman DG, Moher D, Sox H, Riley D (2013). The CARE guidelines: consensus-based clinical case reporting guideline development. Headache J Head Face Pain.

[11] Carnap R (1947). On the application of inductive logic. Philos Phenomenol Res.

[12] Gartlehner G, West SL, Mansfield AJ, Poole C, Tant E, Lux LJ (2012). Clinical heterogeneity in systematic reviews and health technology assessments: synthesis of guidance documents and the literature. Int J Technol Assess Health Care.

[13] Page MJ, McKenzie JE, Bossuyt PM, Boutron I, Hoffmann TC, Mulrow CD, et al (2021). The PRISMA 2020 statement: an updated guideline for reporting systematic reviews. BMJ 71 10.1136/bmj.n71 PMC8005924 33782057

[14] Rafiei A, Hakky TS, Martinez D, Parker J, Carrion R (2014). Superficial dorsal vein injury/thrombosis presenting as false penile fracture requiring dorsal venous ligation. Sex Med.

[15] Al-Reshaid RA, Madbouly K, Al-Jasser A (2010). Penile abscess and necrotizing fasciitis secondary to neglected false penile fracture. Urol Ann.

[16] Aminu S, Usman F, Kyriacos A (2011). A physical sign of coital rupture of superficial dorsal vein of penis. Cent European J Urol.

[17] Eken A, Acil M, Arpaci T (2014). Isolated rupture of the superficial vein of the penis. Can Urol Assoc J..

[18] Dias-Filho AC, Fregonesi A, Martinez CAT, Pimentel ES, Riccetto CLZ (2020). Can the snapping sound discriminate true from false penile fractures? Bayesian analysis of a case series of consecutively treated penile fracture patients. Int J Impot Res.

[19] Nehru-Babu M, Hendry D, Ai-Saffar N (1999). Rupture of the dorsal vein mimicking fracture of the penis. BJU Int.

[20] El-Assmy A, El-Tholoth HS, Abou-El-Ghar ME, Mohsen T, Ibrahiem EHI (2010). False penile fracture: value of different diagnostic approaches and long-term outcome of conservative and surgical management. Urology.

[21] Truong H, Ferenczi B, Cleary R, Healy KA (2016). Superficial dorsal venous rupture of the penis: false penile fracture that needs to be treated as a true urologic emergency. Urology.

[22] Baran C, Topsakal M, Kavukcu E, Karadeniz T (2011). Superficial dorsal vein rupture imitating penile fracture. Korean J Urol.

[23] Nicely ER, Costabile RA, Moul JW (1992). Rupture of the deep dorsal vein of the penis during sexual intercourse. J Urol.

[24] Sharma GR (2005). Rupture of the superficial dorsal vein of the penis. Int J Urol.

[25] Gontero P, Muir GH, Frea B (2003). Pathological findings of penile fractures and their surgical management. Urol Int.

[26] Chung CH, Szeto YK, Lai KK (2006). “Fracture” of the penis: a case series. Hong Kong Med J.

[27] Naraynsingh V, Raju GC (1985). Fracture of the penis. Br J Surg.

[28] Bachoo S, Batura D (2021). Fractures of the penis. Br J Hosp Med.

[29] Koifman L, Cavalcanti AG, Manes CH, Filho DR, Favorito LA (2003). Penile fracture - experience in 56 cases. Int Braz J Urol.

[30] Fedel M, Venz S, Andreessen R, Sudhoff F, Loening SA (1996) The value of magnetic resonance imaging in the diagnosis of suspected penile fracture with atypical clinical findings. J Urol. 155(6):1924–7 8618289

